# Tactile Flow Overrides Other Cues To Self Motion

**DOI:** 10.1038/s41598-017-01111-w

**Published:** 2017-04-21

**Authors:** Laurence R. Harris, Kenzo Sakurai, William H. A. Beaudot

**Affiliations:** 1 Centre for Vision Research, York University, 4700 Keele St., Toronto, Ontario M3J 1P3 Canada; 2 Department of Human Science, Tohoku Gakuin University, 2-1-1 Tenjinzawa, Izumi-ku, Sendai, Miyagi 981-3193 Japan; 3 Division of Human Informatics, Graduate School of Tohoku Gakuin University, 2-1-1 Tenjinzawa, Izumi-ku, Sendai, Miyagi 981-3193 Japan; 4 KyberVision Japan LLC, 5-2-8 Takamori, Izumi-ku, Sendai, Miyagi 981-3203 Japan

## Abstract

Vestibular-somatosensory interactions are pervasive in the brain but it remains unclear why. Here we explore the contribution of tactile flow to processing self-motion. We assessed two aspects of self-motion: timing and speed. Participants sat on an oscillating swing and either kept their hands on their laps or rested them lightly on an earth-stationary surface. They viewed a grating oscillating at the same frequency as their motion and judged its phase or, in a separate experiment, its speed relative to their perceived motion. Participants required the phase to precede body movement (with or without tactile flow) or tactile flow by ~5° (44 ms) to appear earth-stationary. Speed judgments were 4–10% faster when motion was from tactile flow, either alone or with body motion, compared to body motion alone (where speed judgments were accurate). By comparing response variances we conclude that phase and speed judgments do not reflect optimal integration of tactile flow with other cues to body motion: instead tactile flow dominates perceived self-motion – acting as an emergency override. This may explain why even minimal tactile cues are so helpful in promoting stability and suggests that providing artificial tactile cues might be a powerful aid to perceiving self-motion.

## Introduction

It is important to know about our position and movement in space in order to maintain stability and to interact with the world. There are several sources of sensory information that contribute to how we do this. The most obvious are retinal flow and information about acceleration coming from the vestibular system. But other senses can theoretically also be useful, for example reference to a stationary sound source^1, 2^. However, when stability is threatened our first instinct is to act to prevent a fall, which often involves holding on to something to provide stability and obtain somatosensory cues about what is happening. Vestibular-somatosensory interactions play an important role in posture control^3, 4^ where pressure on the skin supplements the vestibular system to provide information about the position of the body relative to the support surface. Indeed, it has become common to refer to the “vestibular/somatosensory” system when discussing orientation perception because of the difficulty of dissociating between the contributions of these two systems. Providing a stable tactile reference point can assist balance^5–8^. Although interactions between the vestibular and somatosensory systems are pervasive^9–12^, the function of this interaction outside posture control, and even precisely how tactile information is actually used for posture control, is far from clear^13^.

Dynamic tactile cues may be involved in monitoring and controlling self-motion through the use of tactile flow. Placing the finger tips lightly on an oscillating bar induces body sway in a standing participant^14^, which suggests a direct role for this kind of tactile flow in assessing body motion. Tactile flow can be created, for example, by airflow over exposed skin surfaces, especially on the face, during movement. Sensing air flow is important to birds, which will start flying when airspeed reaches a certain magnitude^15^, and has been shown to enhance the perception of self-motion in humans^16^. Tactile flow can also be obtained by running the fingertips along a stationary surface while walking. Of course touching surface with the hands is a fairly unusual behavior in our urban existence, although small children like to run their hands along the wall when they walk in a hallway. If vision is not available however, either through blindness or by lack of light, feeling one’s way becomes critical. Movement through the undergrowth of a forest could have provided similar information naturally to the skin of our ape ancestors. Indeed, many cells in the intra-parietal region of the monkey brain, an area thought to be critically involved in the perception of self-motion, receive substantial somatosensory information as well as vestibular and visual input^17, 18^. Cells in this region often have large tactile receptive fields and are directionally selective for movement across the skin reminiscent of the large receptive fields of cells that process optic flow^19, 20^. Indeed the optimum direction for visual and tactile flow for these intra-parietal cells are aligned^17^. Although the intra-parietal region is responsive to translational movement^21^, the somatosensory responses of cells that are responsive to linear acceleration have not been explored. Taken together, these observations suggest that tactile flow may contribute to our sense of self-motion.

Experiments using an array of static vibrators embedded in a seat and activated sequentially to produce tactile apparent motion from front to back over the buttocks modulate magnitude estimates of the speed of a radiating optic flow pattern^22, 23^ implying a visual/tactile interaction during self motion although it is not quite clear which natural movement, if any, this was intended to simulate. Here we used sinusoidal lateral linear motion of the body coupled with natural tactile flow on the fingertips. Visual flow and vestibular cues are combined optimally in the perception of orientation^24^ and in the perception of heading^25^. Might tactile flow be similarly integrated?

Optimal integration of two redundant cues produces a performance intermediary between the responses to either cue alone and also reduces the variance of judgments compared to the variance when either cue is available alone^26^. We therefore compared self-motion perception produced by body motion alone, self-motion perception signaled by tactile cues presented to a stationary observer, and self-motion perception produced by body motion in the presence of simultaneous tactile cues. We measured the perceived time of reversal of sinusoidal oscillatory movement and the perceived speed of movement.

## Results

### Phase Experiment

**Figure 1:**
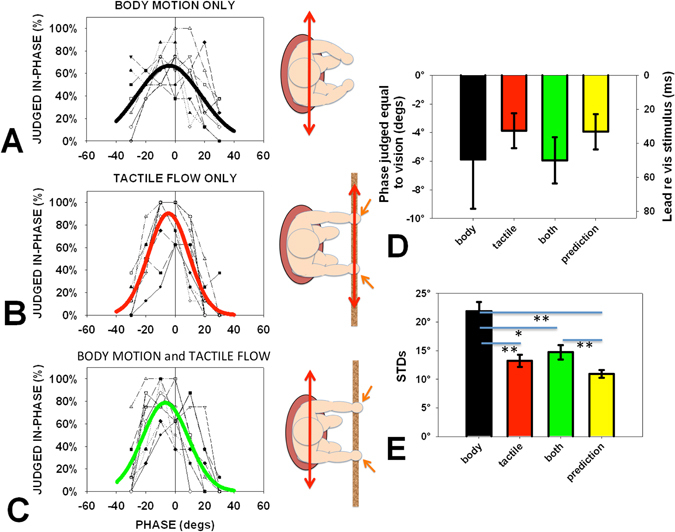
Frequency at which participants chose “same phase” plotted as a function of the phase difference between the visual stimulus and either the body motion only (**A**), tactile flow only (**B**), or combined conditions (**C**). Each participant’s performance is shown, with best-fit Gaussians fitted to the means plotted as a thick line through each data set. The experimental conditions are shown as insets. For the body motion only condition the observer sat on a moving swing in darkness. For the tactile flow only condition the observer held their fingertips lightly over a moving surface. For the combined condition observers oscillated on the moving swing with their fingertips lightly touching an earth-stationary surface. The mean phase with equivalent time delay (**D**) and standard deviation (STD) (**E**) of the fits to each participant’s data is shown along with the prediction of the maximum likelihood estimation (MLE) model (yellow bars). Error bars are standard errors. Horizontal lines with asterisks indicate statistically significant differences. * p < 0.05, **p < 0.01, ***p < 0.001.

Figure 1(A–C) shows the percentage of “same” judgements for body-only (oscillation in the dark), tactile-flow-only (oscillation of a felt surface) and both body and tactile flow (oscillation with fingertips resting lightly on an earth stationary surface) plotted as a function of the introduced delay between the movement and the motion of a visual reference display. There was no statistical difference between the peak phases that were judged as occurring at the same time as the phase reversals of the visual reference motion across the three conditions (Fig. 1D). The mean value was −5.2° ± 2.1°, equivalent to the phase reversal of the visual stimulus preceding the perceived time of phase reversal of the seat’s movement, the tactile movement, or the combined movement by 44 ms ± 18 ms. That is, the visual reference movement was processed about 44 ms slower than either body movement, tactile flow, or both. The standard deviations of the Gaussian fits were significantly different (repeated measures ANOVA: *F*(2, 18) = 14.286, *p* < 0.001, *η*_*p*_^*2*^ = 0.614). Tactile flow alone settings were more precise than for the body alone condition (±13.2° vs ±21.9°, *p* = 0.006). Judgements during body motion in the presence of tactile flow were significantly more precise than in the body motion alone condition (±14.7° vs ±21.9°, *p* = 0.013). The values for the mean Gaussian fits are given in Table 1 and plotted in Fig. 1E.

**Table 1:** The parameters of the best fit Gaussians for all conditions.

Phase judgments	Body	Tactile	Body + tactile	Prediction
Phase Match	−5.9° ± 3.4°	−3.9° ± 1.2°	−5.9° ± 1.6°	−3.9° ± 1.2°
Standard Deviation	21.9° ± 1.6°	13.2° ± 1.0°	14.7° ± 1.3°	11.0° ± 0.7°
Variance	503 ± 76 deg^2^	185 ± 31 deg^2^	231 ± 37 deg^2^	124 ± 16 deg^2^

The numbers are the averages of the fits to each individual’s data. Standard errors are given.

### Modelling

The means of the Gaussians were not significantly different from each other. These values therefore could not be used to provide a clue as to how tactile cues were combined with other body motion cues to produce the overall performance. However, the standard deviations of the judgments were different and we therefore converted them to variance estimates (the square of the standard deviations σ) and compared performance to the predictions of an optimum integration model based on maximum likelihood estimation (MLE)^27^ using the formula:

1 $$1/{{\sigma }_{{\rm{c}}{\rm{o}}{\rm{m}}{\rm{b}}}}^{2}=1/{{\sigma }_{{\rm{b}}{\rm{o}}{\rm{d}}{\rm{y}}}}^{2}+1/{{\sigma }_{{\rm{t}}{\rm{a}}{\rm{c}}{\rm{t}}{\rm{i}}{\rm{l}}{\rm{e}}}}^{2}$$

The mean phase judgments should combine proportional to their weights according to

2 $${{\rm{Phase}}}_{{\rm{comb}}}={{\rm{phase}}}_{{\rm{tactile}}}\ast {{\rm{weight}}}_{{\rm{tactile}}}+{{\rm{phase}}}_{{\rm{body}}}\ast {{\rm{weight}}}_{{\rm{body}}}$$

where the weights are inversely proportional to their variances:

3 $${{\rm{Weight}}}_{{\rm{tactile}}}={{{\rm{\sigma }}}_{{\rm{body}}}}^{2}/({{{\rm{\sigma }}}_{{\rm{body}}}}^{2}+{{{\rm{\sigma }}}_{{\rm{tactile}}}}^{2})$$

4 $${{\rm{Weight}}}_{{\rm{body}}}={{{\rm{\sigma }}}_{{\rm{tactile}}}}^{2}/({{{\rm{\sigma }}}_{{\rm{body}}}}^{2}+{{{\rm{\sigma }}}_{{\rm{tactile}}}}^{2})$$

The predictions of this model for the bimodal condition were obtained for each participant’s data using the body-only and tactile-only conditions. The values are given in Table 1 and in the histograms of Fig. 1D and E where they can be compared to the bimodal data.

A repeated measure ANOVA was conducted to test for differences in the variance between the three conditions (body, tactile, and both). A significant main effect was found, *F*(1.223, 11.008) = 12.164, *p* = 0.004, *η*_*p*_^2^ = 0.575. The variance for the condition when both cues were available was indeed reduced compared to the body-alone condition (*t*(9) = 3.453, *p* = 0.007). However, the variance was not less than when only the tactile cue was present (231 deg^2^ vs 185 deg^2^ for tactile alone) and did not show the reduction in variance (improved precision) that would be expected if the cues were optimally integrated (231 deg^2^ vs the predicted 124 deg^2^ p = 0.007).

### Speed Experiment

**Figure 2:**
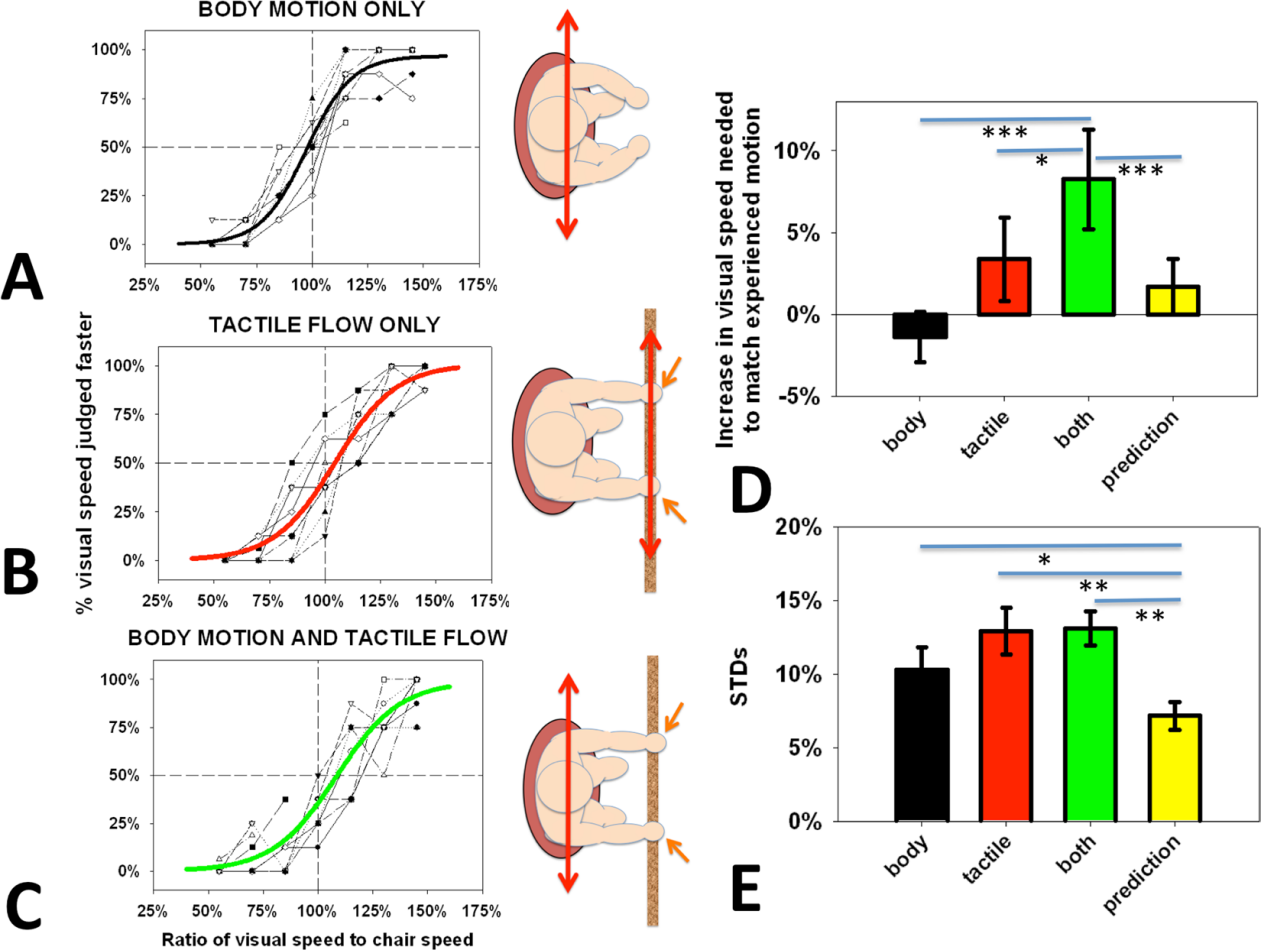
Speed judgments of the peak velocity of body, tactile or combined motion plotted as a function of the ratio of the visual speed to swing’s speed. Data are shown for each participant with a best-fit sigmoid fitted to the means for body motion only (**A**), tactile flow only (**B**), and both (**C**). Sigmoids were fit to each participant’s data and the mean increase in the points of subjective equality (PSE) at which the visual movement matched the experienced motion (**D**) and standard deviations (**E**) are plotted with standard errors and compared to the predictions of the optimum integration model (yellow bars). Horizontal lines with asterisks indicate statistically significant differences. * p < 0.05, **p < 0.01, ***p < 0.001.

Figure 2 (A–C) shows participants’ performance in judging whether a visual stimulus was moving faster or slower than body, tactile or combined motion as a function of the ratio of the two speeds. The point of subjective equality (PSE) is when the speed of the visual motion is perceived as equal to the speed of the seat’s motion and the standard deviation (STD) is a measure of the steepness of the function. A PSE of 100% corresponds to accurate performance. Logistic functions were fitted to the data (see methods). The difference in PSEs from 100%, and STDs are presented in Table 2 and Fig. 2D and E where a positive value indicates that a faster visual speed was needed to match the experienced motion. A repeated measure ANOVA was conducted to test for differences in the speeds between the three conditions (body, tactile, and both). A significant main effect was found, *F*(2, 16) = 10.644, *p* = 0.001, *η*_*p*_^2^ = 0.571. The visual speed that was matched to the body’s motion was significantly faster when tactile cues were present (*MD* = −11.2%, *SE* = 1.84, *p* = 0.001) but not different from tactile alone. Body-only and tactile-only judgments were not different from accurate but when combined, self-motion was matched to a significantly faster visual motion than tactile alone (MD = 6.4% faster, SE = 2.02, *p* = 0.04). There were no significant differences between the standard deviations of any of the curves.

**Table 2:** PSEs and STDs of the ratio of visual speed to body, tactile, or combined motion needed for them to be judged as equal.

Speed judgments relative to 100%	Body	Tactile	Body + tactile	Prediction
PSE (%)	−1.4 ± 1.6	3.4 ± 2.7	9.7 ± 2.4	1.7 ± 1.7
STD (%)	10.3 ± 1.6	12.9 ± 1.7	13.1 ± 1.3	7.2 ± 0.9

Values are the averages of the fits to each individual participant’s data with standard errors.

### Modelling

The variances of the body-only and tactile-only data were used to calculate the prediction for the perceived speed and expected variance of the combined stimulus using equations 3–5 above. Both the variance and PSE for the combined motion were significantly different from the prediction (PSE *t*(8) = 5.045, *p* = 0.001; STD *t*(8) = 4.572, *p* = 0.002). The STD of the combined judgments was not reduced relative to the STD of either component alone and the PSE of the combined judgment did not fall between the judgments for body or tactile motion alone.

**Figure 3:**
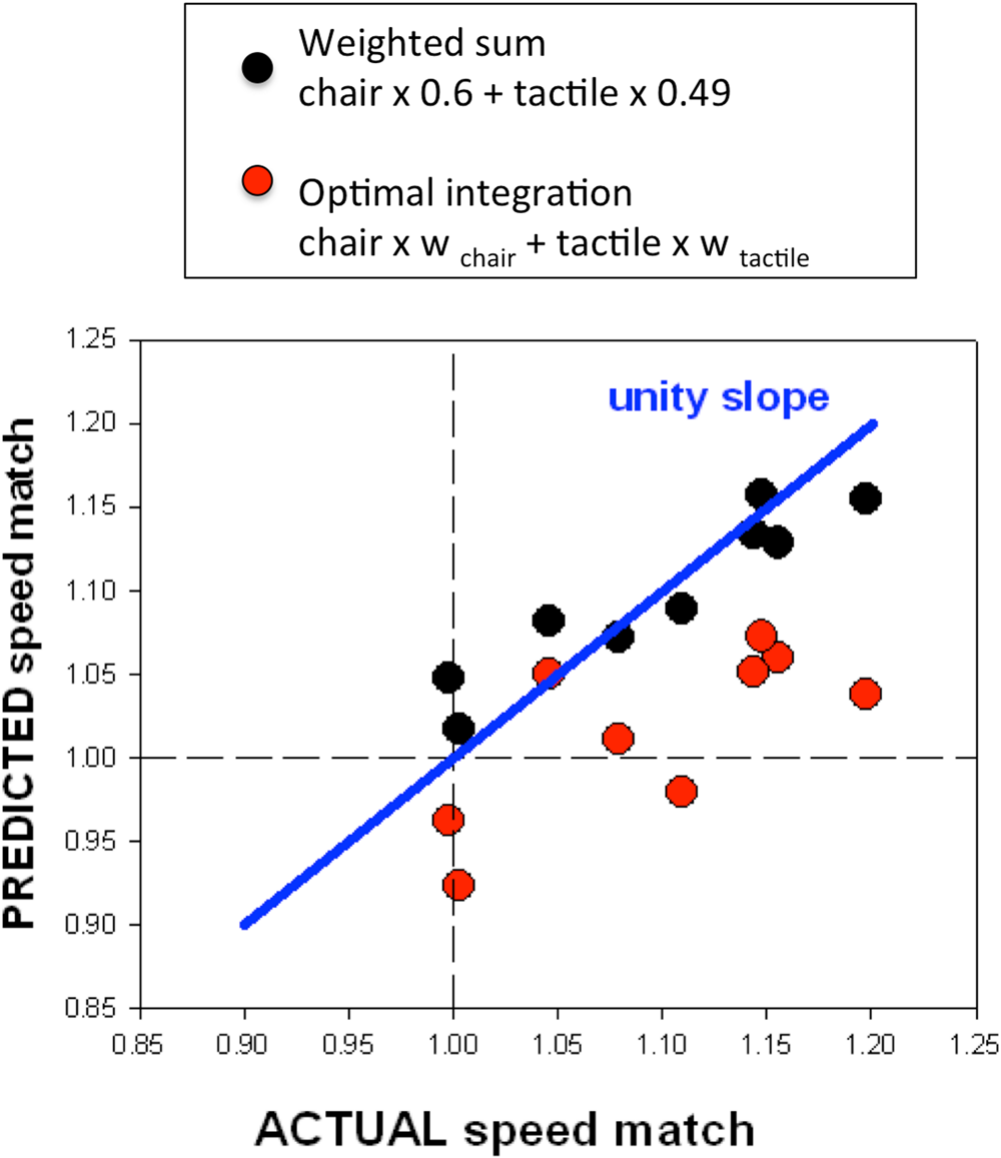
Shows the predicted speed match for each participant based on the optimum integration model fit for each participant (red symbols). The black symbols are the predicted combined speed based on an unconstrained weighted sum model. The solid line has a slope of unity, which would indicate a perfect prediction.

Curiously, there was a correlation between the MLE-predicted speed match and the actual combined speed match (red symbols in Fig. 3, *r* = 0.73, *p* = 0.025) even though the perceived speed of the combined stimulus was consistently faster than the prediction. We fitted a simple weighted sum model with constant weightings for all participants:

5 $${{\rm{speed}}}_{{\rm{comb}}}={{\rm{speed}}}_{{\rm{tactile}}}\ast {{\rm{weight}}}_{{\rm{tactile}}}+{{\rm{speed}}}_{{\rm{body}}}\ast {{\rm{weight}}}_{{\rm{body}}}$$

The best-fit weights were body = 0.6 and tactile = 0.49 (total 1.09, *r* = 0.94, *p* < 0.001) and hence the predicted combined speed was larger than either of the contributors. Figure 3 compares this model with the optimal integration prediction for each participant’s data.

## Discussion

Tactile flow obtained through the fingertips during passive self-motion significantly improved the precision of the perceived timing of body motion, as assessed by matching the timing of the phase reversal of visual motion with tactile motion, body motion or combined motion. Tactile flow also increased the perceived speed of motion when the speed of a visual grating moving with them was matched to their speed induced by tactile motion, body motion, or combined tactile and body motion. However, body and tactile flow cues to self motion were not optimally combined – the variances of timing and speed judgments were not reduced below the variances of either cue used on its own, and the judgments of speed and timing were not made more accurate by the presence of tactile flow.

### Perceived Timing

When comparing the moment at which the experienced motion reversed relative to a visual standard, the presence of tactile information from the fingertips appeared to capture the sensation and improve the precision with which the judgments were made: but not beyond that of the perception of tactile motion only. The improved precision in timing when tactile information was available may be related to an enhancement of spatial attention resulting from an interaction between the vestibular and somatosensory systems^11, 28^.

### Perceived Speed

When both cues were present, participants required faster visual motion to match their perceived motion compared to body motion alone. This was not compatible with an optimal combination of cues but suggests a multiplicative effect such that the total gain (perceived/body motion) becomes 1.09 and overall self-motion is perceived as faster. This phenomenon could be viewed as cues enhancing the tactile signal or tactile cues enhancing the interpretation of body motion. Interactions between the vestibular and somatosensory systems have been shown to modulate the gain of somatosensory responses^10^ leading to enhanced tactile sensitivity^11, 12^. The enhanced speed of perceived motion in the presence of tactile cues could represent a similar multiplicative gain enhancement.

As participants were sitting in the racing car seat (see methods), somatosensory cues were present from their points of contact with the seat (buttocks, back, sides, and feet). These cues contributed to the sense of “body motion” when the seat moved especially because the motion was sinusoidal with a continuously changing acceleration. All the body motion cues (including the seat cues) were therefore in conflict with the fingertip tactile cues in the tactile only condition. Comparing the body motion only (with seat cues) to the combined combination (still with the same seat cues) shows an improvement of precision for timing judgements (Fig. 1E) and a significant increase in perceived speed (Fig. 2E) that can only be due to the additional tactile cues from the finger tips (since the body motion cues, including from the seat were the same in both conditions).

## Conclusion

We conclude that tactile flow is not combined with other cues to motion of the body in a statistically optimal way unlike the combination of visual and vestibular cues to determine heading^29, 30^ or orientation^31^, but instead tactile flow seem to dominate or even enhance perceptual judgments when it is present. We suggest that tactile flow provides an emergency override over other available cues. Our results suggest that providing additional tactile information about self motion through tactile vibrators, for example mounted in a vest^32, 33^, may be useful to enhance or even replace vestibular cues.

## Methods

### Participants

Each experiment had ten participants. For the first experiment (5 female, mean age 32.7 ± 15.8 years) these included the three authors, for the second (6 female, mean age 32 ± 16 years) LRH was not available but the other authors participated. Eight out of the ten participants took part in both experiments. Participants wore corrective glasses as needed. All participants signed an informed consent form and all experiments were approved by the Tohoku Gakuin University Board of Ethics and were performed in accordance with the Treaty of Helsinki.

### Body Motion

A parallel swing was used to provide body motion. The swing had a racing car seat mounted on its base frame. The base frame was suspended from 2.28 m long vertical arms. Oscillatory movement was provided by a programmable stepping motor connected to the swing by an eccentric shank that drove the swing at 0.33 Hz with a constant amplitude of ±10 cm. To measure the movement of the swing, a stylus was attached to the base frame. The stylus moved over a Wacom Intuos 3 tablet positioned on the floor beneath the apparatus to provide the position signal. The swing’s movement pattern was the same for all the conditions of the experiment.

### Visual Stimulus

The visual stimulus was presented on a 27” LED Cinema Display (2560 × 1440 pixels, 60 Hz refresh rate, mean luminance of 190 cd/m^2^) mounted on the swing at a viewing distance of 25 cm. The display was driven by a MacBook Pro (15” Mid-2012 model with a NVIDIA GeForce GT 650 M graphics card) running Psykinematix software (GPU Edition 2.0 build 1113 from KyberVision Japan LLC, Sendai, psykinematix.com). The visual stimulus was a patch of vertically oriented visual grating (sf 0.1 c/d, contrast 50%, 72° horizontal by 68° vertical) that oscillated in the first experiment at the same frequency and amplitude (in cm) but with a variable delay of between −0.25 and 0.25 secs. At zero delay the visual motion was equal and opposite to the movement of the swing simulating an earth-stationary visual stimulus. In the second experiment, the visual grating oscillated at the same frequency as the swing, 180° out of phase with the swing’s motion but with a variable peak velocity of between 11.5 and 31.5 cm/s. The peak velocity of the swing was held constant at 21 cm/s.

### Tactile Stimulus

The tactile stimulus was provided by a flat beam that could be either attached to the moving swing or to an earth-stationary mount. The beam was 2 cm wide and extended beyond the swing in both directions. The beam was covered with a cloth to provide a rough texture when the fingertips were rested lightly on it.

### Conditions

There were three conditions: body motion only, tactile motion only, and both. The three conditions were run in a counterbalanced block design. For the body-motion-only condition participants sat in the racing car seat with their hands on a palm rest in front of them that was fixed to the swing. For the combined condition the earth-stationary beam was positioned at a comfortable distance and height so that the participant could rest their fingertips on it while placing their wrist on the seat-stationary palm rest. For the tactile-motion-only condition, participants sat on an earth-stationary chair facing an earth-stationary screen with the oscillating beam placed in the same position as for the combined condition relative to both them and the screen. The apparatus was arranged next to the swing to which the beam was attached. Thus the beam moved exactly the same amount relative to the participant as in the combined condition. All conditions were performed in a dark room with the apparatus shrouded in a black cloth so that the only thing visible was the screen. The screen was fixed to the swing and moved with the participant. The screen thus did not provide any information as to their movement.

### Procedure

Participants sat in the racing car seat in one of the three arrangements described above. Participants viewed the visual grating and judged their own movement or the movement of the beam relative to the visual stimulus. There were eight trials for each phase or speed judgment (with seven levels of phase from −30° to +30° and seven speeds from 11.5 cm/s to 31.5 cm/s). They made their responses verbally in either English or Japanese: either SAME or DIFFERENT for the phase judgments, or FASTER or SLOWER for the speed judgments.

The instantaneous position of the swing was recorded for one complete cycle of its sinusoidal motion prior to visual stimulus onset so as to synchronize the movement of the grating with the movement of the swing and to allow the insertion of an appropriate phase lead or lag. Each trial had a duration of 9 seconds: the visual stimulus was presented for 2 cycles of the oscillation (i.e., 6 seconds) followed by a blank period of 3 seconds during which the swing continued to oscillate while the participant responded. If no response was provided within the 3 s, that condition was repeated at a later point in the sequence.

### Data Analysis

#### PHASE

The frequency of “same” judgments was plotted as a function of the phase difference between the body or tactile motion for each participant and the visual motion and fitted with a Gaussian:

6 $${\rm{freq}}={\rm{a}}\ast \exp \,(-0.5\ast {(({\rm{x}}-{{\rm{x}}}_{0})/{\rm{\sigma }})}^{2})$$

where x_0_ is the phase at the peak of the Gaussian and σ is the standard deviation. Variance is the square of the standard deviation, σ^2^.

#### Speed

The percentage of “faster” judgments was plotted against the speed of the visual stimulus for each participant and fitted with sigmoidal logistic function:

7 $${\rm{Percentage}}={\rm{a}}/(1+\exp \,(-({\rm{x}}-{{\rm{x}}}_{0})/{\rm{\sigma }}))$$

where x_0_ is the point of subjective equality (PSE) and σ is the standard deviation. Outliers were identified as more than 2 STD from the mean. One participant was unable to perform the speed judgment task at all and was removed from the analysis. Five data points (out of 189) from four participants were removed by this criterion before logistics were fit.

### Statistical Analysis

Repeated measures analyses of variances (ANOVAs) were used for statistical analyses, with alpha set at *p* < 0.05. Post-hoc multiple comparisons were made using Bonferroni corrections. Greenhouse-Geisser correction was used when the assumption of sphericity was not met. Pearson correlations and paired *t*-tests were two-tailed, with alpha set at *p* < 0.05.

### Convention

For the phase experiment, 0° corresponds to the visual stimulus being 180° out of phase (earth stationary). A positive phase then corresponds to the visual display lagging the motion of the body. Since the frequency of the swing’s motion was fixed at 0.33 Hz, each degree of phase lag corresponds to 8.4 ms.

For the speed experiment, the peak visual stimulus speed is expressed as a fraction of the peak speed of the swing. Thus a PSE of 1 corresponds to when they were judged equal (i.e., both 21 cm/s) and greater than 1 means that the visual stimulus had to be moving faster than body, tactile or combined motion in order to be judged as moving at the same speed.
