# Erratum: Tactile Flow Overrides Other Cues To Self Motion

**DOI:** 10.1038/s41598-017-04864-6

**Published:** 2017-07-21

**Authors:** Laurence R. Harris, Kenzo Sakurai, William H. A. Beaudot

**Affiliations:** 1 Centre for Vision Research, York University, 4700 Keele St., Toronto, Ontario M3J 1P3 Canada; 2 Department of Human Science, Tohoku Gakuin University, 2-1-1 Tenjinzawa, Izumi-ku, Sendai, Miyagi 981-3193 Japan; 3 Division of Human Informatics, Graduate School of Tohoku Gakuin University, 2-1-1 Tenjinzawa, Izumi-ku, Sendai, Miyagi 981-3193 Japan; 4 KyberVision Japan LLC, 5-2-8 Takamori, Izumi-ku, Sendai, Miyagi 981-3203 Japan

*Scientific Reports* **7**:1059; doi:10.1038/s41598-017-01111-w; Article published online 21 April 2017

The original version of this Article contained an error in the title of the paper, where the word “Overrides” was incorrectly given as “Overides”. This has now been corrected in the PDF and HTML versions of the Article.

In addition, the original HTML version of this Article omitted an affiliation for Kenzo Sakurai. The correct affiliations are listed below:

Department of Human Science, Tohoku Gakuin University, 2-1-1 Tenjinzawa, Izumi-ku, Sendai, Miyagi, 981-3193, Japan.

Division of Human Informatics, Graduate School of Tohoku Gakuin University, 2-1-1 Tenjinzawa, Izumi-ku, Sendai, Miyagi, 981-3193, Japan.

KyberVision Japan LLC, 5-2-8 Takamori, Izumi-ku, Sendai, Miyagi, 981-3203, Japan.

This error has now been corrected in the HTML; the PDF version was correct from the time of publication.

